# Association of IL-10 (− 1082 A/G) and IL-6 (− 174 G/C) gene polymorphism with type 2 diabetes mellitus in Ethiopia population

**DOI:** 10.1186/s12902-021-00738-1

**Published:** 2021-04-15

**Authors:** Birhanu Ayelign, Markos Negash, Henok Andualem, Tadelo Wondemagegn, Eyuel Kassa, Tewodros Shibabaw, Yonas Akalu, Meseret Derbew Molla

**Affiliations:** 1 Department of Immunology and Molecular Biology, School of Biomedical and Laboratory Science, College of Medicine and Health Sciences, University of Gondar, Gondar, Ethiopia; 2 Department of Medical Laboratory Science, College of Medicine and Health Sciences, Debretabor University, Debretabor, Ethiopia; 3 University of Gondar Comprehensive Specialized Hospital, Gondar, Ethiopia; 4 Department of Biochemistry, School of Medicine, College of Medicine and Health Sciences, University of Gondar, Gondar, Ethiopia; 5 Department of Physiology, School of Medicine, College of Medicine and Health Sciences, University of Gondar, Gondar, Ethiopia

**Keywords:** Interlikun-6, Interleukin-10, Type 2 diabetes mellitus, Northwest Ethiopia

## Abstract

**Background:**

Interleukin (IL)-6 and IL-10 are the most important cytokine with pro and anti-inflammatory activities, respectively. Dysregulation of IL-6 and IL-10 are associated with increased risk of developing Type 2 Diabetes Mellitus (T2DM). Despite this, a fundamental understanding of both cytokine gene polymorphisms with its expression is critical in understanding of cellular mechanism of insulin resistance as well as T2DM intervention. Therefore, this study aimed to assess IL-6 (− 174 G/C) and IL-10 (− 1082 A/G) gene polymorphism, and its association with T2DM, North West Ethiopia.

**Methods:**

A comparative cross-sectional study from January to May 2018 was conducted on study participants with T2DM and apparently healthy controls. Deoxyribonucleic acid (DNA) extraction and genotyping was carried out by using amplification refractory mutation system polymerase chain reaction to detect polymorphism of IL-6 and IL-10 gene at the position − 174 and − 1082, respectively. The logistic regression model was fitted to assess the association of between cytokine gene polymorphisms and T2DM. Odds ratio with 95% CI was determined to assess the presence and strength of association between the explanatory variables and outcome variable. A *P*-value < 0.05 was considered as statistically significant.

**Result:**

Participants carrying the GG genotype of IL-6 (− 174) (OR (95% CI) *=* 4.61 (2.07–10.54) was a high likelihood of having T2DM compared to those carrying the CC and AA genotypes. AA and AG genotypes of IL-10 (− 1082) were at lower odd of developing T2DM compared to those carrying the GG genotype. In addition, individuals carrying the G allele of IL-6 (− 174) have 2.82-fold odds of developing T2DM compared to individuals carrying the C allele **(**OR (95% CI) =2.81 (1.78–4.50)).

**Conclusion:**

Our study revealed that genetic polymorphisms of IL-6 (− 174) GG genotype is the potential host genetic risk factors to T2DM. While, IL-10 (− 1082) AA genotype is negatively associated with T2DM. Therefore, IL-6 (− 174) and IL-10 (− 1082) genetic variation may be considered as a biomarker for early screening and diagnosis of T2DM.

**Supplementary Information:**

The online version contains supplementary material available at 10.1186/s12902-021-00738-1.

## Background

Type 2 Diabetes Mellitus (T2DM) is a group of metabolic syndrome characterized by hyperglycemia associated with the defect of insulin action [1, 2]. Globally, the number of people with diabetes mellitus (DM) has been increased by four folds in the past three decades as a result of urbanization and associated lifestyle change [3]. According to international diabetes federation (IDF), there are 463 million people with diabetes in 2019 in the world, and this number is expected to increase up to 700 million by 2045 [4]. About 1 in 11 adults worldwide now has diabetes mellitus, around 90% of them have T2DM, and 75% of DM patients are living in developing countries [5]. It has been expected that the fastest increase of patients living with T2DM will found to be in Sub Saharan Africa (SSA) countries in the next two decades [6]. Although the incidence and prevalence rate of T2DM is increasing in Africa, the proportion of undiagnosed DM in Africa, which is around 69% is extremely higher than that of developed countries (37%) [7]. The profile of diseases in the African continent has changed from infectious diseases such as malaria, tuberculosis and HIV to chronic noncommunicable diseases, including T2DM. According to 2017 report, the prevalence of diabetes in Ethiopia is found to be 5.2% among adults with a total of 2.6 million cases of DM in the country; of this, T2DM accounts the highest proportion [8, 9]. A recent study finding also revealed that the proportion of hidden T2DM is high among adults who are living in the urban areas of Northwest Ethiopia which is 6.34% [10]. Furthermore, the medical expense of DM patients is about three times higher than the general population without diabetes [3].

The major threat of T2DM patients is associated with micro and macro vascular complications, which is considered as the major cause of morbidity and mortality [11]. Recently, low level of chronic inflammation has been found to have great implication on the onset and progression of T2DM [12, 13]. Studies have shown that high level of inflammatory and anti-inflammatory cytokines such as IL-6 and IL-10 are detected in plasma of patients with T2DM, and thus, associated with its complication [14–17]. Interlikun-6 is a pleiotropic cytokine mainly produced by T cells and macrophages, mapped to human chromosome 7p15–p21 [18]. It regulates a wide range of immune activities, production of cell adhesion molecules, acute phase protein, and facilitates the release of other cytokines in response to inflammatory stimuli [19]. In addition, it affects glucose homeostasis and metabolism directly, or indirectly by acting on muscle cells, hepatocytes and pancreatic β cells [20]. In fact, different ethnic populations have different levels of variability in single nucleotide polymorphisms (SNPs). Hence, polymorphism within the coding and non-coding region of cytokine genes cause interindividual variation in their expression, leading to difference in immune responses [21] and may eventually lead to increased risk of infection and occurrence of a number of chronic diseases [22]. Recent studies revealed that there is a link between IL-6 promoter polymorphism (− 174 G/C) and circulating IL-6 levels with respect to the development of several diseases, however, the effect of IL-6 gene polymorphism with the development of T2DM is not clearly understood despite the presence of contradictory findings [23–25]. While scholars have been found that IL-6 -174 G/C polymorphism is associated with the susceptibility and individuals are the greatest risk of developing T2DM [20], others have still assumed no association [26]. Moreover, studies also showed − 174 G/C polymorphism can serve as a predictor for the progression of complications, particularly kidney diseases in T2DM patients [27, 28] and for developing comorbidity such as tuberculosis [29]. However, few studies reported that there is no association between this polymorphism with diabetes associated kidney diseases [26, 30].

Interleukin-10 (IL-10) is a multifunctional cytokine with both immunosuppressive and anti-angiogenic functions [31]. The human IL-10 gene is located on chromosome 1q31–32, a locus genetically linked to susceptibility to a number of autoimmune diseases, including T2DM [32, 33]. The replacement of nucleotide A by G on the promoter region of the IL-10 gene (− 1082 A/G) will increase the transcription of both genes, approximately two folds, as a result it increases the production of this cytokine. Due to this fact, it is expected that the polymorphic genotype may be associated with an increased frequency of T2DM [21, 34]. Now a day, the burden of the T2DM rapidly increases worldwide, especially in middle and low-income countries including Ethiopia. With an increasing number of T2DM patients in Ethiopia, knowledge on genetic background and marker, which contribute to the etiology and progression of T2DM is important in the intervention and managements of the disease. To date, as a controversial, it has been difficult to determine the exact relationship between IL-6 and 10 genotypes and the corresponding cytokine production with respect to T2DM. Therefore, the aim of this study was to investigate the association of cytokine gene polymorphisms (IL-6 (− 174 G/C) and IL-10 (− 1082 A/G)) with T2DM.

## Materials and methods

### Study design and period

A cross-sectional study was conducted from January to May 2018 in adult patients with T2DM compared to control populations living in northwest Ethiopia.

### Study participants and sample processing

A total of 150 participants (75 laboratory-confirmed T2DM patients and 75 controls were selected using a convenient sampling method. The diagnosis of type 2 diabetes is based on the guidelines of the American Diabetes Association and the WHO [35, 36]. Participants with confirmed DM or newly diagnosed diabetes using fasting plasma venous glucose of ≥7 mmol/l (126 mg/dl) or random plasma venous glucose of ≥11.1 mmol/l (200 mg/dl) were included. The control groups were confirmed by fasting plasma venous glucose or random plasma venous glucose test to be free of T2DM. For both groups with a history of coronary artery disease and/or other metabolic disorders, malignant tumors and/or autoimmune diseases and less than 18 years of age were excluded from the study. In addition, control groups having with the family history of diabetes, use of hypoglycemic drugs were excluded from the study.

The sample size of the study was calculated using G power version 3.1.9.4 software by selecting chi-square test. We considered that alpha = 0.05, power (1- β) = 0.8 (80%), effect size (d) = 0.3 and df = 5. Then, the sample size becomes 143 for both groups, and by considering 5% of non-response rate the total study participants was 150, with 1:1 ratio of case control group.

### Data collection and variables

Data were collected through semi-structured interviewer administered questionnaires. The questionnaires were prepared by national language (English), then translated to local language (Amharic) and re-translated back to English to check the consistency. They were developed based on the related literatures of previously published papers [37] and collected by experienced nurses. Moreover, the questionnaire includes socio-demographic and clinical variables (S1).

### Blood sample collection and biochemical test analysis

About 5 ml of whole blood were collected with a serum separator tube from each study participants. Of this, 2 ml of blood were utilized for blood glucose determination. Serum was obtained through centrifugation at 3000 rpm for 7 min using Rotanta 960 centrifuge for blood glucose measurement. Then, blood glucose test was done or serum was stored at − 20 °C in sterile circumstances at immunology and molecular laboratory until the analysis was done. The blood glucose level was measured by an enzymatic test in the human (German) A25 Biosystem kit. Consequently, participants were classified as case or control groups based on their blood glucose level, which was categorized according to the American Diabetes Association and WHO guidelines on DM diagnosis. In order to maintain the overall quality of laboratory analysis, standard operating procedures at the pre-analytical, analytical and post-analytical stages of laboratory services were addressed. The collection and testing of the blood samples was carried out by laboratory technologists.

### DNA extraction and genotyping

Genomic DNA was extracted from whole blood using salting out method [38]. The quality of pure, integral and intact genomic DNA was estimated by Nano drop using A260/A280 absorbance ratio between 1.8 to 2.0 indicate that high quality of DNA [37, 39, 40]. *A PCR-RFLP technique was used to amplify IL-6 (− 174 G/C) polymorphism based on the protocol given by Sery* et al. *(2003). The primer sequences used to amplify 204 bp PCR products are given in* Table 1*. The PCR reaction was performed in a total volume of 50* μl *containing 200 ng of genomic DNA, 200* μM *of each dNTP, 50 mM KCl, 1 mM each of the primers and 1.5 U of Taq polymerase. RFLP of IL 6 was performed in a total reaction volume of 15 μl, where 10 μl of 204 bp PCR product, 1.8 μl of buffer, 8 U of TaqI restriction enzyme and 3 μl of distilled water were mixed together and incubated for 5 h at 65 °C. The digested product was analyzed by electrophoresis with 3% agarose gel containing ethidium bromide. The 204 bp product was cleaved in two fragments of 180 and 24 bp by Taq I enzyme. The uncut product of 204 bp identified CC and the cut fragment identified GG genotype. The heterozygous GC genotype was identified by the presence of both 204 and 180 bp products.* IL-10 (− 1082 A/G) gene were amplified and genotyped using thermo cyclic PCR in a 15 μl reaction mixture containing 100 ng of template DNA, buffer (100 mM Tris, pH 9.0; 500 mM KCl; 15 mM MgCl2; 0.1% gelatin), 200 μM dNTP, 10 pmol of each primer and 1.0-unit Taq DNA polymerase. Each reaction employed a generic antisense primer and one of the two allele-specific sense primers, which is found in the Table 1 below. The amplified product was evaluated with 2% agarose gel stained with ethidium bromide and then UV light gel documentation system was used to observe the band [38, 41].

**Table 1:** Primers for amplifications of IL-6-174G/C and IL-10-(-1082) by polymerase chain reaction, 2018

Genetic polymorphism	Primer sequence	Annealing C^0^	Amplicon size
IL-6-174G/C	Forward primer		GG-180 bp, GC-204 and 180 bp CC-204 bp
	5′-ACTTTTCCCCCTAGTTGTTTCTTTC-3’	60	
	Reverse primer		
	5′-AGACATGAGCCTCAGACATCTCAGT-3’		
IL-10-(−1082)	Sense IL-10-(− 1082) G		
	5′-CTACTAAGGCTTCTTTGGGAG-3’	56	161
	Sense IL-10-(−1082) A		
	5′-ACTACTAAGGCTTCTTTGGGAA-3’		161
	Antisense IL-10-(−1082)		
	5′-CAGTGCCAACTGAGAATTTGG-3′.		

Footnote: *bp* base pair, *TM* Temperature.

### Statistical analysis

The data were double entered and cleaned using Epi Data 3.1 (Jens M. Lauritsen & Michael Bruus) and exported to SPSS version 20 (IBM, New York, and U.S). Data was collected, summarized, tabulated and analyzed. *Kolmogorov-Smirnov and Shapiro-Wilk test was applied to determine the distribution of the data. Student t-test was applied where the data was normally distributed and Mann-Whitney test was applied where the data was not normally distributed.* Chi square test was used to test the deviation from Hardy-Weinberg Equilibrium (HWE) of SNPs by comparing the observed and expected frequencies. The association between IL-6 (− 174) and IL-10 (− 1082) gene polymorphism with T2DM was determined by using the Odds ratio (OR) and 95% confidence intervals from logistic analyses. A *P* value < 0.05 was considered as statistically significant.

## Result

### Socio-demographic characteristics

A total of 150 age-sex matched case-control participants were included in this study. Majority (82*/150)* of participant were male as well the Mean ± SD age of both study subjects were 55 ± 96 and 55 ±96, respectively. Looking in to educational status, *52% of T2DM subjects studied secondary school and above followed by primary school attendants which accounted 25.3%; whereas the occupational status profile showed that merchants accounted the greatest part (*48.0%*) followed by civil servants (*37.3%*) *(Table 2)*.*

**Table 2:** Socio-demographic profiles of the study participants, North West Ethiopia, 2018 (*n* = 150)

Variables	T2DM, N (%)	Health control, N (%)	Total N (%)	*P*-value
*Sex*				
*Male*	41(54.7)	*41(54.7)*	82 *(*54.6*)*	0.48
*Female*	34 (45.3)	*34 (45.3)*	68 *(*45.4*)*	
Age in years (Mean **±** SD)	55 ± 96	55 ± 96		
Residency				
Rural	*15 (20)*	25 *(*33.3*)*	40 *(*26.7*)*	0.42
Urban	*60 (80)*	50 *(*66.7*)*	110 *(*73.3*)*	
Educational status				
*Illiterate*	13 (17.3)	15 (20)	28 (18.7)	
*Primary school*	19 (25.3)	29 (38.7)	48 (32)	0.51
*Secondary school and college*	39 (52)	22 (29.3)	61(40.7)	
*Degree and above*	4 (5.3)	9 (12.)	13 (8.7)	
Occupational status				
Farmer	7 (9.3)	7 (9.3)	14 (9.3)	
Merchant	36 (48.0)	30 (40)	66 (44)	0.79
Civil servant	28 (37.3)	25 (33.3)	53 (35.3)	
Student	2 (2.7)	5 (6.7)	7 (4.7)	
Other	2 (2.7)	8 (10.7)	10 (6.7)	

### Allelic association of IL-6 and IL-10 polymorphism with T2DM

We performed an analysis of allele and genotype frequencies of IL-6 (− 174) G/C and IL-10 (− 1082) G/A gene polymorphisms. The frequency of IL-6 allele G in T2DM patients and control groups were 64 and 39%, respectively (OR (95% CI) =2.82 (1.78–4.50)). Furthermore, the frequency of IL-10 (− 1082) G allele in T2DM patients and control groups were 77 and 60% (OR (95% CI) =0.49 (0.29–0.78)). Our finding indicates that the alleles G in IL-6 and IL-10 have a significant association with T2DM than allele C and A, respectively as it is more frequently expressed (Table 3).

**Table 3:** Allelic relationship between IL-6 (−174) G/C and IL-10 (−1082) G/A polymorphism and the odds of developing T2DM, North West Ethiopia, 2018 (*n =* 150)

Allele	T2DM (*N* = 75) N (%)	Controls (*N =* 75) N (%)	Fishers exact test	OR (95%CI)
IL-6 (−174)				
G	96 (64)	58 (39)	< 0.001**	2.82 (1.78–4.50)
C	54 (36)	92 (61)		
IL-10 (−1082)				
A	36 (23)	60 (40)	0.004**	0.47 (0.29–0.78)
G	114 (77)	90 (60)		

*T2DM* Type 2 Diabetes Mellitus, *N* Number, *CI* confidence interval, *OR* Odd ratio, ** highly significant (P < 0.001)

### Genotypic association of IL-6 and IL-10 polymorphism with T2DM

*We further analyzed the genotype frequencies of* IL-6 (− 174) G/C and IL-10 (− 1082) G/A gene *polymorphisms between the T2DM and control groups (*Table 4*). The* most frequent genotype in *of* IL-6 (− 174) gen polymorphisms are (GG), followed by GC and CC in T2DM. While t*he* most frequent genotype in IL-10 (− 1082) are (GG), then the heterozygous GA and finally the rare genotype or the one that suffered the mutation (mutated). *Statistical analysis of the* χ2 *test showed a significant difference in* IL-6 (− 174) *(P = 0.001,* OR (95% CI) *=* 4.61 (2.07–10.54) and IL-10 (− 1082) AA genotype distribution *(P = 0.012,* OR (95%CI) *= 0.30* (0.101–0.892) *between the two study groups. Calculated frequencies of genotype and allelic expression of* IL-6 (− 174) and IL-10 (− 1082) *gene polymorphism have been done using Hardy Weinberg equilibriums.* Thus, we found that according to genotype frequency, both the patient and control group significantly deviate from HWE law as shows *P* < 0.05. Therefore, in order to solve the problem, we performed a standard chi-square test on a contingency table.

**Table 4:** Genotypic relationship between IL-6 (-174) G/C and IL-10 (-1082) G/A gene polymorphism and the odds of developing T2DM, North West Ethiopia, 2018 (*n* = 150)

Genotype	T2DM (*N =* 75) N (%)	Controls (*N* = 75) N (%)	*P-*value	OR (95%CI)
IL-6 (−174)				
GG	38 (50.7)	16 (23.3)		4.61 (2.07–10.54)
GC	20 (26.7)	26 (34.7)	0.001*	1.49 (0.65–3.411)
CC	17 (22.7)	33 (44)		- (ref)
IL-10 (−1082)				
AA	6 (8)	12 (16)		0.30 (0.101–0.892)
AG	24 (32)	36 (48)	0.012*	0.40 (0.198–0.808)
GG	45 (60)	27 (36)		- (ref)

*T2DM* Type 2 Diabetes Mellitus*, N* Number, *OR* Odd ratio, *ref reference*, *P significant < 0.05, **P highly significant < 0.001

## Discussion

The purpose of the present study was to investigate the relationship between IL-6 and IL-10 gene polymorphisms with T2DM. Insulin resistance is understood to be the inability of cells to use glucose, and the glucose-reducing effects of insulin are abnormal, leading to hyperglycemia, and T2DM [42, 43]. Scholars have been reported that T2DM is associated with chronic inflammation that could be attributed to dysregulation of the innate immune system and this is a potential link between metabolic syndrome and diabetes [21]. Interleukin 6 is one of the most prominent cross-taking cytokine involved between inflammation and T2DM [44] whereas, IL-10 which is an anti-inflammatory cytokine plays an important role in the regulation of immune response, leading to decreased cytokine production, lower tissue factor expression, inhibiting matrix-degrading metalloproteinase, and promoting the phenotype switching of lymphocytes to the Th2 cell [45, 46]. Moreover, IL-10 has been shown to influence both the susceptibility and course of various diseases, and the different polymorphisms in the IL-10 gene promoter have been associated with T2DM prevalence and severity [21].

In this study, we found that the G alleles of − 174 IL-6 polymorphism are more frequent in T2DM participants than apparently healthy individuals. Hereafter, individuals carrying the G allele of IL-6 (− 174) were 2.82-fold odds of developing T2DM compared to individuals carrying the C allele **(**OR (95% CI) =2.82 (1.78–10.54)). The evidence suggested that the allele G of IL-6 -174G/C was higher in T2DM patients than those of healthy controls. It has been shown that the -174G/C polymorphism of the IL-6 promoter region has a direct effect on gene transcription [47]. Following its expression, IL-6 can function on various immune cells to trigger and/or increase inflammatory response, macrophage activation and fibrosis contributes to end organ damage such as diabetic nephropathy and neuropathy [48, 49]. Similarly, GG genotypes of − 174 IL-6 polymorphism are more frequently observed in T2DM than apparently healthy individuals.

These findings suggested that the IL-6 -174G/C polymorphism might be a risk factor for T2DM among the Ethiopian population though, future studies required to confirm the finding in large scale. Similar observations were found from Indians [50], and Native Americans and Caucasians [51]. In contrast, finding from Brazil showed that genotype and allele frequencies of polymorphisms for cytokine gene IL-6 found no statistically difference between T2DM and control groups [16]*. And again our finding was not supported by a report from Pakistan and Greece that showed IL-6174 allele C was significantly associated with IL-6 expression and T2DM development* [14, 52]*. Besides this, IL-6 -174 GG polymorphism is associated with increased insulin sensitivity and may protect the development of T2DM in Framingham population* [53]*. Therefore, the lack of agreement between different studies could be due to either the variation in ethnic origin or the interaction between the susceptible gene loci and various environmental determinants.*

Interestingly, there was a significant association between IL-10 (− 1082) G/A gene polymorphism and T2DM. The GG genotype was more frequently observed in T2DM patients than in apparently healthy controls that contains AA and AG genotype *(P = 0.012,* OR (95%CI) *= 0.30 (0.101–0.892) and 0.40* (0.198–0.808), respectively). Despite the fact, AA and AG genotypes are 0.30- and 0.40-fold odds of negatively developing T2DM. Our finding provides a clue that the IL-10 (− 1082) G/A polymorphism might be a protective for T2DM among Ethiopian population. In line with this, a study conducted in Egypt and China population showed that the IL-10 gene − 1082, GG genotype was significantly increased the risk of T2DM [54, 55]. In contrast to our finding, a Brazilian study indicated that genotype and allele frequencies of polymorphisms in cytokine IL-10 gene − 1082 G/A found no statistically significant difference between T2DM and control groups [16]. In fact, the causes of T2DM are environmental or genetic factors, the genetic polymorphism of cytokines like IL-6 and 10 caused by up/down regulation of inflammatory cytokines to trigger insulin resistance [21]. In particular, T2DM are often characterized by insulin resistance due to low production or impaired signaling cascade of IL-10 concomitant to enhanced expression of pro-inflammatory molecules.

Overall, IL-10 and IL-6 play an immense role in regulating the immune response. Single nucleotide polymorphism (SNP) at promoter region − 1082 and − 174 of IL-10 and IL-6, respectively able to facilitate binding of many more transcription factors with RNA-polymerase II (RNA-Pol II) to this target gene of interest. Therefore, enhanced gene expression is not only due reaction between promoter region and RNA Pol-II, but also with the involvements of different trans-acting proteins as basal transcription factor. Subsequently, these mutations may play a part in the reaction affinity between the DNA binding domain of the RNA-pol II and promoter, and increase its catalytic activities [56–58]. Therefore, the IL-10 and IL-6 production may alter the concentration of these cytokines in plasma of blood circulation. Therefore, here we can understand that gene polymorphism is one of the most important biomarkers for early screening and diagnosis of both communicable and non-communicable disease. Those aspects are becoming increasingly essential preventive strategic option of T2DM.

The limitation of this study was a small sample size and did not quantify IL-6 and IL-10 in the study groups, may strongly indicate, ether the wild or mutated gene of these cytokines is associated with an increased serum cytokine level in each of the study groups. However, in the study population, the G allele is predicted to increase the expression of IL-6 and 10 in plasma, a significant mediator of insulin resistance that could lead to diabetes through inhibiting insulin receptor tyrosine kinase and interfering with its action on adipocytes, and skeletal muscle (60).

## Conclusion

The genetic polymorphisms of IL-6 and IL-10 in T2DM patients demonstrated higher frequencies of G allele and GG genotypes. Moreover, the polymorphisms of these cytokines were significantly associated with T2DM. This finding suggested that the genetic polymorphism may be contributed to the expression of cytokine, which result insulin resistance and T2DM. Furthermore, the identification of polymorphism of IL-6 and IL-10 gene is used not only for screening purpose, but also as additional diagnostic tools for T2DM.

## Supplementary Information

**Additional file 1.** Questionnaire (English version).

## Data Availability

The data used and/or analyzed during the current study are available from the corresponding author on reasonable request (https://github.com/birhanu42/-Gene-Polymorphism).

## Abbreviations

IL: Interleukin
PCR: Polymerase Chain Reaction
SNP: Single Nucleotide Polymorphisms
TNF-α: Tumor Necrotic Factor alpha
T2DM: Type 2 Diabetic Mellitus
