# Supplementary material for: Association of IL-10 (− 1082 A/G) and IL-6 (− 174 G/C) gene polymorphism with type 2 diabetes mellitus in Ethiopia population
Source: BMC Endocr Disord. 2021 Apr 15;21:70. doi: 10.1186/s12902-021-00738-1 (PMC8051082; doi:10.1186/s12902-021-00738-1)
Supplement: Supplementary file 1 — Additional file 1. Questionnaire (English version). [file 12902_2021_738_MOESM1_ESM.docx]

**Questionnaire (English version)**

**Title of the project:** “**Association of** **IL-10 (-1082 A/G) and IL-6 (-174 G/C) Gene Polymorphism with Type 2 Diabetes Mellitus in Ethiopia population**”

Participant ID------------

| Socio demographic characteristics | | | |
| --- | --- | --- | --- |
| Question | | | Response |
| 1 | Sex | | A. Male  B. Female |
| 2 | Age in year | | --------------Year |
| 3 | Residence | | A. Rural  B. Urban |
| 4 | Educational status | | 1. Illiterate 2. Primary school 3. Secondary school and college 4. Degree and above |
| 5 | Do you have any family member suffering from DM disease? | | 1. Yes 2. No |
| 6 | Are you suffering from kidney disease? | | 1. Yes 2. No |
| 7 | Are you suffering from coronary artery disease? | | 1. Yes 2. No |
| 7 | Do you have any disease other than DM such as hypertension, retinopathy, Cardiovascular problem | | 1. Yes 2. No |
| 8 | If yes, what type of disease do you have and the treatment currently taken? | | 1. Type of disease------------ 2. Type of treatment --------- |
| Laboratory result | | | |
| 5 | | Blood glucose level | ------------- mg/dl |
| 6 | | IL-10 gene polymorphism | Allele  Genotype |
| 7 | | IL-6 gene polymorphism | Allele  Genotype |

Name and signature of the data collector -----------------------------------------------------
